# Salinity Effects on Sugar Homeostasis and Vascular Anatomy in the Stem of the *Arabidopsis Thaliana* Inflorescence

**DOI:** 10.3390/ijms20133167

**Published:** 2019-06-28

**Authors:** Sahar Sellami, Rozenn Le Hir, Michael R. Thorpe, Françoise Vilaine, Nelly Wolff, Faiçal Brini, Sylvie Dinant

**Affiliations:** 1 Institut Jean-Pierre Bourgin, INRA, AgroParisTech, CNRS, Université Paris-Saclay, 78000 Versailles, France; 2 Biotechnology and Plant Improvement Laboratory, Center of Biotechnology of Sfax, (CBS)-University of Sfax, Sfax 3018, Tunisia; 3 University of Sousse, Higher Institute for Agronomy of Chott-Mariem, Sousse 4042, Tunisia; 4 Plant Science Division, Research School of Biology, The Australian National University, Canberra, ACT 0200, Australia

**Keywords:** salt stress, sugar homeostasis, secondary cell wall, acclimation, phloem, xylem, carbon allocation, inflorescence, stem, transport, heterotrophy

## Abstract

The regulation of sugar metabolism and partitioning plays an essential role for a plant’s acclimation to its environment, with specific responses in autotrophic and heterotrophic organs. In this work, we analyzed the effects of high salinity on sugar partitioning and vascular anatomy within the floral stem. Stem sucrose and fructose content increased, while starch reduced, in contrast to the response observed in rosette leaves of the same plants. In the stem, the effects were associated with changes in the expression of *SWEET* and *TMT2* genes encoding sugar transporters, *SUSY1* encoding a sucrose synthase and several *FRK* encoding fructokinases. By contrast, the expression of *SUC2*, *SWEET11* and *SWEET12*, encoding sugar transporters for phloem loading, remained unchanged in the stem. Both the anatomy of vascular tissues and the composition of xylem secondary cell walls were altered, suggesting that high salinity triggered major readjustments of sugar partitioning in this heterotrophic organ. There were changes in the composition of xylem cell walls, associated with the collapse and deformation of xylem vessels. The data are discussed regarding sugar partitioning and homeostasis of sugars in the vascular tissues of the stem.

## 1. Introduction

Soil salinity constitutes a major threat limiting crop growth and productivity worldwide. Around 20% of the cultivated land and more than one-third of irrigated land are affected worldwide by high salinity [1]. Two main phases are observed in the responses to salt stress [2]. The first is a response to the osmotic stress due to the increase of osmotic pressure in the soil that reduces water availability for the plant. The second phase is an ionic stress due to uptake of sodium ions (Na^+^) and their subsequent accumulation in the leaves. This accumulation is detrimental for plant cells and leads to major alterations of the metabolism and to nutritional imbalance [2,3]. Halophytic species, which are salt-tolerant, maintain their development during the second phase of the salt stress, by contrast to salt-sensitive species that usually suffer reduced growth during this ionic phase [2]. Unfortunately, many crops, such as rice (*Oryza sativa*), soybean (*Glycine max*) and maize (*Zea mays*), are not tolerant to salt (glycophytes). A range of species-specific mechanisms has evolved in plants to counteract the adverse effects of salt stress, including salt acclimation [4,5,6]. For example, in the glycophyte Arabidopsis, seedlings can survive up to 200 mM NaCl if they are first exposed to a low level of salt stress [7], indicating that the responses to salt stress may be alleviated by a period of acclimation. Various responses to high salinity have been described, including in roots the exclusion of Na^+^ or their sequestration in the vacuole [2,8,9]. Another relevant feature is the adaptive modification of carbon allocation and sugar metabolism. For example, a reduction of Carbon allocation towards the roots was observed in tomato plants (*Lycopersicon esculentum* L. cv. Momotarou), even before photosynthetic activity of the leaves had declined [10]. Similar effects on carbon translocation via the phloem have also previously been reported in tobacco [11]. A consequence of such changes in carbon allocation is a higher accumulation of sugars in the mature leaves under high salinity, as seen in tobacco, rice, poplar, tomato and cotton [11,12,13,14,15,16], the degree of sugar accumulation depending on a leaf’s age [12,17]. Changes in sugar metabolism and homeostasis have also been reported, for example in ryegrass, wheat, tomato or cotton [12,16,18,19], with sugars acting as osmo-protectants [3,20]. Changes in sugar homeostasis are also potentially associated with modifications of cell walls, as in red-osier dogwood (*Cornus stolonifera*), poplar (*Populus canescens* and *Populus euphratica*) and coffee (*Coffea arabica*) [21,22,23], acting either to stiffen cell walls and reduce the entry of salt, or to increase cell wall elasticity and maintain cell turgor by allowing increased cell volume as solutes enter [24]. However, sugar homeostasis and transport are highly regulated during the development of the plant, as are cell wall viscoelastic properties, and so there could be changes in plant susceptibility to stresses that depend on carbon status [25,26].

In *Arabidopsis thaliana*, studies of the response to high salinity have mostly examined the vegetative stage [7,27,28,29,30,31,32,33], with limited attention to the reproductive phase [34,35]. However, the inflorescences contribute more than the rosette to lifetime carbon gain [36,37], which in turn is critical for seed production [38]. The physiology of the floral stem during its development is marked by a succession of heterotrophic (sink) then exporting (source) phases, associated with different developmental phases leading to flowers and siliques formation [39]. Here, we focus on the reproductive stage, concerning sugar transport, homeostasis and partitioning in response to high salinity. There were changes in both the sugar content and vascular anatomy in the stem, associated with changes in the expression of genes involved in sugar transport and metabolism. The composition of the secondary cell wall of the stem xylem was also affected.

## 2. Results

### 2.1. Growth of the Rosette and Sugar Accumulation in the Rosette Leaves under High Salinity Vary over Time

**Figure 1:**
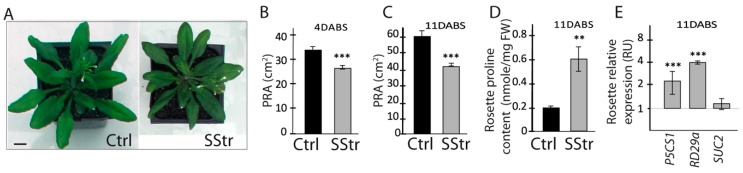
Effect of salt stress on rosette leaves: their growth and the expression of several stress markers. (**A**) Rosette growth at 4 days after the beginning of salt treatment (DABS) under control (Ctrl) and salt-stress (SStr) treatments. Bar: 1 cm (**B**,**C**) Projected rosette area in Ctrl and SStr plants at 4 DABS (**B**) or 11 DABS (C) (*n* ≥ 10 and *n* = 6, respectively at 4 and 11 DABS). (**D**) Proline content in the rosette mature leaves in Ctrl and SStr plants at 11 DABS (*n* ≥ 5). (**E**) Relative expression of *P5CS1*, *RD29a* and *SUC2* in the rosette mature leaves in Ctrl and SStr plants at 11 DABS. RU: relative unit. The accumulation of mRNA for each gene in response to salt stress was normalized by the mean value of Ctrl plants (*n* = 4–6) and expression data are shown on a log2 scale. Values represent means ± *SE*, in Ctrl (black bar) and SStr plants (grey bars). Asterisks indicate significant difference of treatments compared to control plants (** *p* < 0.01; *** *p* < 0.001).

To study the effects of high salinity during the reproductive stage, the plants were maintained in standard growth conditions during the vegetative stage and plants were transferred, one day before the onset of the floral bud, to a high salinity but with a concentration (150 mM NaCl) that Arabidopsis plants can withstand [34,40]. In these conditions (Figure 1A), the projected rosette area (PRA) after 4 and 11 days of salt treatment (SStr) was reduced by 22% and 31%, respectively, compared to the Ctrl plants, respectively (Figure 1B,C). Another effect of salt treatment was a higher accumulation of proline in the rosette leaves of SStr plants at 11 days after the beginning of salt treatment (DABS) compared to Ctrl plants (Figure 1D), a typical response to salt stress. In agreement with these data, we observed a higher accumulation of the transcripts of *P5CS1*, which encodes the ‘delta1-PYRROLINE-5-CARBOXYLATE SYNTHASE 1’, a key enzyme of the proline biosynthesis [41] and of ‘*RESPONSIVE TO DESSICATION 29a*’ gene (*RD29a*), another stress marker [42]. However, salinity had no effect in the rosette leaves on the transcript level of *SUC2* (*SUCROSE TRANSPORTER2*) that encodes the main phloem-loading sucrose transporter in Arabidopsis (Figure 1E), indicating that the capacity of the rosette to maintain carbon allocation to sink organs is maintained.

**Figure 2:**
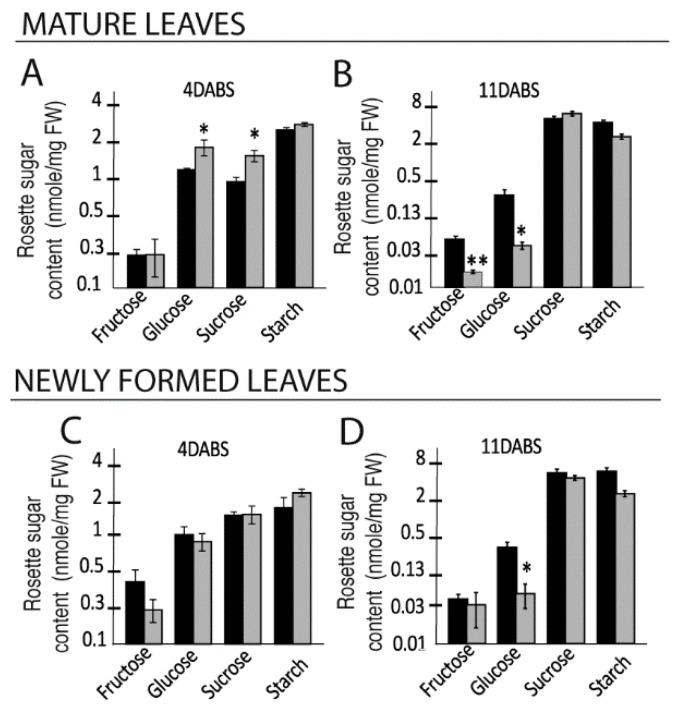
Variations in soluble sugars and starch content in rosette leaves in response to salt treatment. (**A**–**D**) Soluble sugar and starch contents in Ctrl and SStr plants, shown as bar plots drawn on a log2 scale. Contents in mature (**A**,**B**) and newly formed leaves (**C**,**D**) at 4 DABS (**A**,**C**), and 11 DABS (**B**,**D**). Values represent means ± *SE* (*n* = 4 to 6), in control (black bar) and stress plants (grey bars). Asterisks indicate significant difference of treatments compared to control plants (* *p* < 0.05; ** *p* < 0.01).

We also examined the accumulation of soluble sugars in mature and newly-formed rosette leaves (Figure 2). At four days after the beginning of salt treatment (DABS), glucose and sucrose content was higher in the mature leaves of stressed plants compared to control plants (Figure 2A), while at 11 DABS, fructose and glucose content in SStr plants was significantly lower than in Ctrl plants (Figure 2B). In the younger leaves that had emerged during salt treatment, sugar content at 4 DABS was similar in Ctrl and SStr plants (Figure 2C) but by 11 DABS the glucose content was lower, just as for mature leaves at that stage (Figure 2B,D). There was no effect of salt treatment on starch content in either mature or young leaves, whenever they were sampled (Figure 2).

### 2.2. Physiological Responses of the Stem under High Salinity

**Figure 3:**
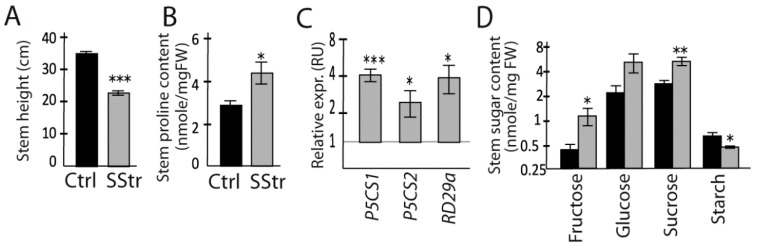
Responses in the stem to high salinity. Growth, proline content, sugar content and gene expression in the stem of Ctrl and SStr plants, with black bars for Ctrl plants and grey bars for SStr plants. (**A**) Stem height. (**B**) Proline content. (**C**) Relative expression (relative expr.) of *P5CS1*, *P5CS2*, and *RD29a*. For each gene, the relative transcript accumulations were normalized by the mean value of control plants. (**D**) Sugar and starch contents. Values represent means ± *SE* (*n* = 5–6 except for sugar and starch content *n* = 4–6). Asterisks indicate significant difference of treatments compared to control plants (* *p* < 0.05; ** *p* < 0.01; *** *p* < 0.001).

The experiment was set up in such a way that the stem developed only after the beginning of salt treatment, while the rosette could provide C resources that accumulated in normal growth conditions and beyond. At 13 DABS, flowers and the first siliques emerged on the stem. At this stage, the stem height of salt-treated plants was reduced by 35% in SStr plants compared to the Ctrl plants (Figure 3A). As expected for a salt treatment, the accumulation of proline was higher in the stem of SStr plants compared to Ctrl plants (Figure 3B). Accordingly, in response to high salinity, we observed in the stem of SStr plants a higher accumulation of *P5CS1*, *P5CS2* and *RD29a* transcripts (Figure 3C), with fold-changes consistent with other reports of long-term responses to salt treatment [7,30,43].

Fructose and sucrose contents were significantly higher in the stem of SStr plants compared to Ctrl plants (Figure 3D), with a tendency for a higher accumulation of glucose. The total of soluble sugars, i.e., hexoses plus sucrose, was higher in SStr plants compared to Ctrl plants (Table 1). By contrast, the starch content was lower (Figure 3D). The relative values of hexoses, sucrose and starch were also analyzed in order to obtain information on the source or sink status of the stem at the stage of the analysis. Indeed, the hexose-to-sucrose ratio can be used as an indicator of physiological status [44,45], high values being observed in sink organs, while the sucrose-to-starch ratio can be used as an indicator of leaf aging and source strength [46], being greater in younger expanding or old organs than in source organs.

The rosette leaves and the stems showed contrasting responses. The hexoses-to-sucrose ratio reduced after salt stress in both mature and young leaves, while the sucrose-to-starch ratio increased (Table 1). Both observations are consistent with a change in sugar metabolism, likely associated with leaf aging and a decline of the photosynthetic activity, as is expected to occur under high salinity [2]. In the stem, by contrast, there was no effect on the hexose-to-sucrose ratio, although that ratio was twenty-fold higher than in the leaves, indicating that the stem is a strong sink at this stage, in both conditions. This higher value for the sucrose-to-starch ratio for the stem is also characteristic of sink tissues, undergoing growth and expansion.

The total soluble sugar content was much higher in the stem of the plants under high salinity compared with control conditions (Table 1). This increased accumulation of sugars is important for maintaining osmotic pressure when Na^+^ and Cl^−^ ions accumulate in the vacuole [2]. Because the storage of sugars in the stem at a heterotrophic stage has not been examined in detail, we examined the expression of genes that are involved in sugar partitioning.

### 2.3. Transcriptional Profiling of Stem Genes Involved in Sugar Transport and Metabolism under Higher Salinity

**Figure 4:**
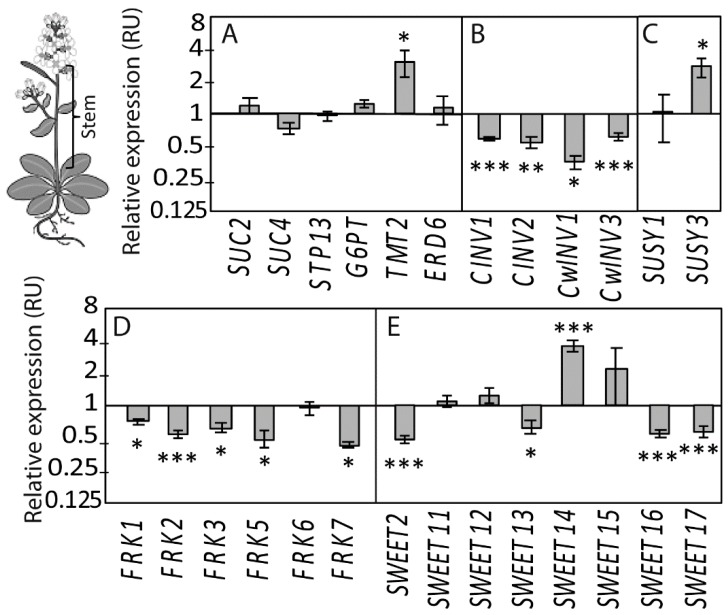
Relative expression of candidate genes in the stem under high salinity in the stem of Ctrl and SStr plants at 13 DABS. For each gene, bar plots showed the mean accumulation of transcripts ± *SE* (*n* = 5–6) normalized by the mean value of Ctrl plants, with Ctrl plants on the left side (black bars) and SStr plants on the right side (grey bars). (**A**–**E**) Relative expression of genes coding for (**A**) disaccharide and monosaccharide transporters (*SUC2*, *SUC4*, *STP13*, *G6PT*, *TMT2*, *ERD6*), (**B**) cytosolic and cell wall invertases (*CINV1*, *CINV2*, *CwINV1*, *CwINV3*), (**C**) sucrose synthases (*SUSY1* and *SUSY3*), (**D**) fructokinases (*FRK1*, *2*, *3*, *5*, *6* and *7*), and (**E**) SWEET sugar transporters (*SWEET2*, *11*, *12*, *13*, *14*, *15*, *16*, *17*). Asterisks indicate significant difference of treatments compared to control plants (* *p* < 0.05; ** *p* < 0.01; *** *p* < 0.001).

We first selected a subset of candidate genes associated with sugar metabolism and transport using in silico approaches and exploiting the available transcriptomic databases. We used the expression profiles generated in response to osmotic stresses in the BAR database [47], exploiting a dataset that was obtained in the shoot or roots of young seedlings treated for 24 h by cold, heat, drought or salt treatments (Supplementary Figure S1). We selected the *TONOPLAST MONOSACCHARIDE TRANSPORTER2* (*TMT2*), *SUGAR TRANSPORT PROTEIN 13* (*STP13*), *GLUCOSE-6-PHOSPHATE/PHOSPHATE TRANSLOCATOR 2* (*G6PT*/*GPT2*) and *EARLY RESPONSE TO DEHYDRATION 6* (*ERD6*) genes that are coding for monosaccharide transporters, eight *SUGARS WILL EVENTUALLY BE EXPORTED* (*SWEET*) genes, six *FRUCTOKINASE* (*FRK*) genes, two *SUCROSE SYNTHETASE* (*SUSY*) genes, two *CYTOSOLIC INVERTASE* (*CINV*) genes and two *CELL WALL INVERTASE* (*CwINV*) genes (Figure 4).

**Figure 5:**
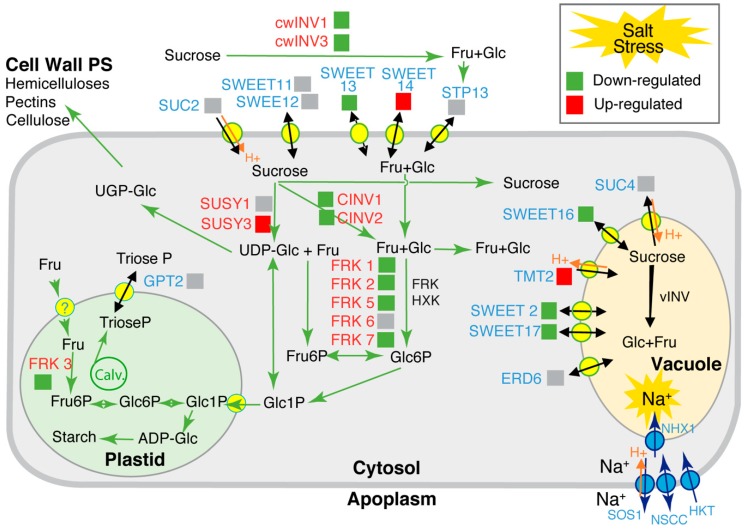
Scheme summarizing the expression data for genes involved in sugar transport (*TMT2*, *SWEET2*, *SWEET13*, *SWEET14*, *SWEET16*, *SWEET17*) and sugar homeostasis (*cwINV1*, *cwINV2*, *SUSY3*, *CINV1*, *CINV2*, *FRK1*, *FRK2*, *FRK3*, *FRK5* and *FRK7*) in the heterotrophic stem tissue under high salinity. The figure shows changes in transcript accumulation in plants submitted to high salinity compared to controls. Red squares indicate genes upregulated in response to salt stress and green squares correspond to downregulated ones. Gray squares indicate genes with no significant changes. In blue letters: transporters and facilitators; in red letters: enzymes of the carbohydrate central metabolism. The main products of the corresponding transporters or enzymes are shown. FRK: fructokinase. HXK: hexokinase. SUSY: sucrose synthase. CINV: cytosolic invertase. cwINV: apoplasmic invertase. vINV: vacuolar invertase. UDP-Glc: UDP-glucose. Fru: fructose. Glc: glucose. ADP-Glc: ADP-Glucose. Glc1P: Glucose-1P. Glc6P: Glucose-6P. Fru6P: Fructose-6P. Calv. Calvin Benson Cycle. PS: polysaccharides. In response to high salinity, Na^+^ is transported from the apoplasm to the cytosol by NSCC channels and HKT transporters and in the vacuole by NHX1 Na^+^/H^+^ antiporters (blue circles), while the Na^+^/H^+^ antiporters SOS1 allow exclusion of Na^+^ across the plasma membrane [8,49,50].

We added to this selection *SUC2* and *SUC4* that code for sucrose transporters that can be affected by salt stress [48]. We examined the effect of the salt treatment for transcript accumulation of these genes in stem tissue (Figure 4). Comparing SStr to Ctrl plants, there was no significant effect on *SUC2*, *SUC4*, *STP13*, *G6PT*/*GPT2* or *ERD6*, but *TMT2* increased significantly (Figure 4A). Lower transcript accumulation was observed for *CINV1*, *CINV2*, *CwINV1* and *CwINV3* (Figure 4B). Higher transcript accumulations were found for *SUSY3* in SStr plants compared to Ctrl (Figure 4C). A lower accumulation of transcripts was observed for *FRK1*, *FRK2*, *FRK3*, *FRK5* and *FRK7* (Figure 4D) and for *SWEET2*, *SWEET13*, *SWEET16* and *SWEET17* (Figure 4E), while a higher accumulation was observed for *SWEET14*. The expression data are summarized in Figure 5 with respect to the function of these genes.

### 2.4. High Salinity Alters the Anatomy of the Stem Vascular Tissues

**Figure 6:**
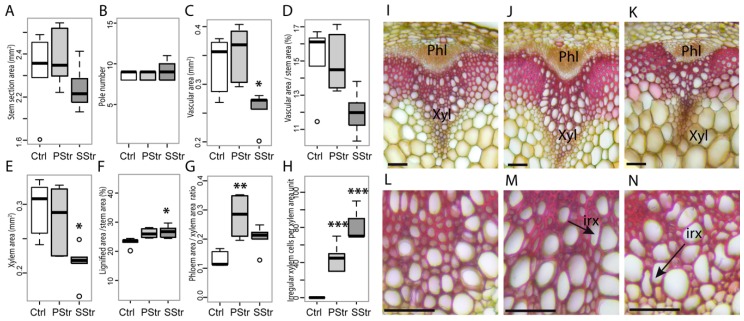
Stem anatomy in response to high salinity. Growth and anatomy of the stem of Ctrl, PStr and SStr plants, with black bars for Ctrl plants, light grey bars for PStr plants and dark grey for SStr plants. In (**A**) to (**H**), the box and whisker plots show the distribution of the biological replicates (*n* = 4 to 6). Inside black lines represent medians; the top and bottom ends of the boxes represent the first and the third quartiles, respectively; whisker extremities represent maximum and minimum data points and open circles represent outliers. (**A**) Area of the stem cross section. (**B**) Number of vascular bundles per stem section. (**C**) Total area of the vascular bundles per section. (**D**) Proportion of the vascular bundle area per total stem section area, expressed as a percentage. (**E**) Total xylem area per section. (**F**) Proportion of the lignified area (including interfascicular fibers and xylem) per total stem section area, expressed as a percentage. (**G**) Phloem area-to-xylem area ratios. (**H**) Number of irregular xylem cells-to-xylem section area ratio. **A** to **H**: *, ** and *** indicate significant difference of treatments compared to Ctrl plants (* *p* < 0.05; ** *p* < 0.01; *** *p* < 0.001). (**I**–**N**) Transversal cross section of basal stem stained with Safranin O and Alcyan Blue for Ctrl plants (**I**,**L**), Sstr plants (**K**,**N**) and PStr plants (**J**,**M**). Black arrows indicate the irx cells. Xyl: Xylem, Ph: Phloem. Bar = 50 µm.

*FRK1*, *2*, *3*, *5* and *7* genes, for which we observed a downregulation under high salinity, play a role in the development of the vascular tissues [51]. Moreover we recently showed that a salt treatment applied three days before the appearance of the floral bud induces changes in the organization of the vascular tissues [35], an effect that was dampened in plants that had first been submitted to a salt pretreatment. To examine the relationships between gene expression and anatomy, we examined in detail the anatomy of the stem of Ctrl and SStr plants. We also included in this study plants submitted to a progressive pre-treatment of 25 mM and 50 mM NaCl before the 150 mM salt stress (Pre-Treatment before Stress, hereafter named “PStr plants”) (Figure 6).

In SStr plants compared to Ctrl plants, we observed a slight variation of the stem section area and no change in the number of vascular bundles, compared to control plants (Figure 6A,B). The total vascular area in the sections was smaller, with a reduced proportion of vascular area and xylem area per stem section (Figure 6C–E). By contrast, the total lignified area was higher (Figure 6F), potentially participating to the mechanical stability of upright stems. In PStr plants, we observed limited changes compared to Ctrl plants, except that the phloem-to-xylem areas ratio was higher in the PStr plants compared to SStr and Ctrl plants (Figure 6G). We also observed deformed and collapsed xylem vessels in both PStr and SStr plants, a feature that corresponds to a phenotype known as “irregular xylem” (irx) found in mutants deficient in secondary cell wall formation [52]. The fraction of xylem vessels that was irregular was higher in SStr plants than in PStr plants (Figure 6H). Imaging of the vascular bundles further showed a reduction of the xylem area in SStr plants, and the occurrence of irx vessels under high salinity, a phenotype that was more pronounced in SStr plants than in PStr plants (Figure 6I–N).

Principal component analysis (PCA) (Supplementary Figure S2A,B) and hierarchical clustering analysis (HCA) (Supplementary Figure S2C) were applied to the whole dataset, including gene expression, stem anatomy and sugar, starch and proline contents. We observed a clear separation of the three treatments (Ctrl, SStr, PStr) within the projection on the two first principal component planes (Supplementary Figure S2A,B). The clustering analysis further indicated similar patterns in the xylem-to-phloem ratios, the expression of *FRK1*, *2*, *3*, *5* and *7*, and the expression of *CwINV1* and *3* (Supplementary Figure S2C). A correlation was observed between the expression of *RD29a* and sugars content, an association previously reported as the primary response to sucrose in seedlings [53]. The sucrose-to-starch ratio was related to the pattern of expression of *SUSY3*. Correlations were found in the expression patterns of *SWEET11* and *SWEET12* (Supplementary Figure S2C). These observations further support the hypothesis that modifications observed in the xylem anatomy may be associated with changes in the expression of *FRK* and *cwINV* genes, and with changes in sugar partitioning. This prompted us to test the effects of high salinity on the composition of the secondary cell wall (SCW) of the xylem.

### 2.5. Effect of Salt Stress with and without Acclimation on the Secondary Cell Wall Composition of the Floral Stem

**Figure 7:**
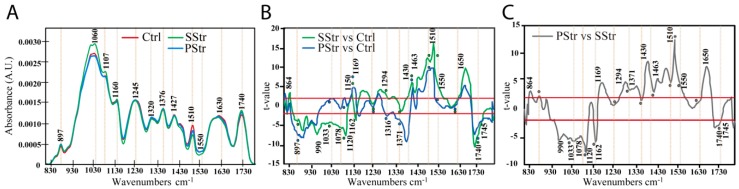
Secondary cell wall (SCW) composition of the stem of plants under high salinity. (**A**) Average FT-IR spectra obtained on the xylem of Ctrl, PStr and SStr plants. Maxima are indicated above the curves. A.U.: arbitrary units. In (**B**,**C**), *t*-values from Student’s *t*-test of pairwise comparisons between absorbances are shown: PStr versus Ctrl (black line), SStr versus Ctrl (green line) in (**B**) and SStr versus PStr (grey line) in (**C**). The red lines are at *t* = +2: *t*-values below −2 or above −2 correspond to significantly stronger and weaker absorbances respectively (*p* < 0.05). Data between the red lines (−2 and +2) correspond to non-significant differences between the different conditions. In (**B**,**C**): Maxima identified on (**A**) are reported as circled dots on the curves. Spectral wavenumbers for cellulose (1033, 1060, 1120 and 1162 cm−^1^) [54,55], cellulose and hemicellulose vibrations (864, 897 and 1169 cm−^1^) [54,55,56,57], xyloglucan (945, 1078, 1316 and 1371 cm−^1^) [54,57], acetylated xylans (1740 cm−^1^) [58,59], other polysaccharides linkages (990, 1150 and 1294 cm−^1^) [54,56], esters (1745 cm−^1^) [55], lignins (1430, 1463 and 1510 cm−^1^) [60,61] and amides I and II (1550 and 1650 cm^−1^) [61] are reported above or below the curves in (**B**) and (**C**).

The secondary cell wall composition was analyzed for stem cross-sections by Fourier-transform infrared spectroscopy (FT-IR) (Figure 7A). Spectral differences were observed between PStr and SStr plants compared to Ctrl plants (Figure 7A). A Student’s *t*-test was used to compare the differences between the average spectra obtained in Ctrl, PStr and SStr plants (Figure 7B,C).

The *t*-values, plotted against each wavenumber of the spectrum (Figure 7B), showed that SStr plants exhibited significant differences at wavenumbers between 880 cm−^1^ and 1122 cm−^1^ compared to Ctrl plants, with several negative peaks (higher absorbance than in the control plants) at 917, 983, 1114 and 1160 cm−^1^. This region of spectra maps to various linkages of the (1,4)-β-D-glucan polymer [55], with the wavenumbers at 990, 1033, 1060, 1120 and 1162 cm−^1^ corresponding to cellulose [54] and wavenumbers at 864, 897, 945, 1078, 1150 and 1169 cm−^1^ corresponding also to hemicelluloses [54,55,56].

By contrast, in PStr plants compared to Ctrl plants, PStr plants had two negative peaks around 897 and 945 cm−^1^ and a positive peak close to 1169 cm−^1^, which correspond to linkages of both cellulose and hemicellulose [54,56] (Figure 7B). Furthermore, spectra of PStr plants also exhibited higher absorbances around 1316 and 1371 cm−^1^ compared to Ctrl plants (Figure 7B), which are also assigned either to cellulose or to hemicellulose [56,57]. Comparison of PStr and SStr spectra confirmed spectral differences between the two conditions with higher absorbances at wavenumbers around 990 cm−^1^, 1033 cm−^1^, 1120 cm−^1^ and 1162 cm^−1^ for SStr plants (Figure 7C), that are being assigned to COC, CO, CC or OCH of cellulose linkages [55], and higher absorbances for PStr around 1169 cm−^1^, 1294 and 1371 cm^−1^, associated to both cellulose and hemicellulose vibrations [54,56,57].

Higher absorbances in both PStr and SStr plants compared to Ctrl plants were found in the spectra region between 1712 cm−^1^ and 1790 cm−^1^ (Figure 7B) which is associated with C=O stretching of xylans [62,63,64,65,66]. Furthermore, spectra of both PStr and SStr plants exhibited lower absorbances around 1430 cm−^1^, 1463 cm−^1^, and 1510 cm−^1^ compared to Ctrl plants (Figure 7B). These wavenumbers correspond to linkages related to lignin [60,61]. Interestingly, spectra of SStr plants exhibited significant differences in their absorbances between 1400 and 1492 cm−^1^, around 1430 cm−^1^, 1463 cm−^1^ and at 1510 cm−^1^ compared to PStr plants (Figure 7C), indicating a more severe effect in lignin content in plants submitted to salt stress with no pretreatment (SStr) compared to plants with a pretreatment (PStr) (Figure 7A,C). Altogether, these results showed that compared to Ctrl plants, cellulose and hemicelluloses compounds vary in the xylem SCW depending on SStr and PStr plants, with variations in acetylated xylans and lignins in both PStr and SStr plants.

## 3. Discussion

### 3.1. Carbon Allocation and Sugar Homeostasis in the Stem under High Salinity

Despite the important role of the Arabidopsis stem to provide nutrients for the development of flowers, siliques and fruits, there are few reports on how sugar metabolism and transport are coordinated in this organ during its development, or on its response to abiotic stresses. Additionally, whether this organ acts as a sugar producer (source) and/or as a sugar consumer (sink) at a given stage of development is critical for any interpretation of variations in sugar homeostasis. Before seeds start filling, the stem can be considered a sink organ, and then it becomes the dominant source [67].

Our study focused on the plant response at 13 DABS, i.e., 12 days after bolting. At this stage, the stem was elongating and the first siliques started to emerge. The stem hexoses-to-sucrose and sucrose-to-starch ratios were higher than in source leaves (Table 1); both are consistent with a strong sink activity in the stem. Sucrose and fructose contents in the stem of salt-treated plants were higher than in control plants (Figure 3), starch content was reduced, while the hexoses-to-sucrose ratio and sucrose-to-starch ratios were higher, consistent with an even greater sink activity. By contrast, in the mature rosette leaves, we did see an early transient accumulation of sucrose and glucose in response to high salinity at 4 DABS, followed by a recovery at 11 DABS (Figure 2), with the 4 DABS-phase of salt stress corresponding potentially to an osmotic response to high salinity [2]. It was not associated with a higher accumulation of starch, revealing that in these growth conditions changes in sugar homeostasis were not sufficient to alter starch accumulation at midday. Because of the recovery at 11 DABS, the source activity of the rosette is likely unaltered at this stage. Interestingly, the expression levels of *SWEET11*, *SWEET12* and *SUC2*, were no different in the stem of salt-treated plants compared to controls. Likewise, there was no change in the *SUC2* expression level in the rosette leaves of salt-treated plants compared to controls. SWEET11, SWEET12 and SUC2 are the main actors in phloem loading [68,69,70]. SUC2 also acts in the retrieval of leaked sucrose [71] while SWEET11 and SWEET12 have been proposed to participate in the stem to the export of sugars to adjacent tissues during their formation [72]. Altogether, these data indicate that, rather than a change in carbon allocation in response to salt stress, associated with modification of the sugar loading rate, sugar metabolism and partitioning changed in the stem, with higher accumulation of sugars. De novo synthesis and storage of sugars, which often occurs in glycophytes in response to an osmotic stress, has a high energetic cost, added to the cost of sequestering Na^+^ in vacuoles [73,74]. However, because salt stress would lead over time to the reduction of photosynthesis, the higher accumulation of sugars in the stem could instead represent a transient storage of carbon to be utilized when fixation declines. Such a strategy would be adaptive for sustainable supply of carbon to the seeds. How these changes in sugar partitioning can impair seed yield and quality is not known. The use of metabolic indicators, such as hexoses-to-sucrose and sucrose-to-starch ratios might also be relevant in predicting plant fitness.

Our findings also raise the question of the function of accumulated hexoses and sucrose under salt stress in the stem of *Arabidopsis thaliana*. It is frequently stated that sugars act in the cytosol as compatible osmolytes to offset the sequestration of Na^+^ in the vacuole [75]. However, in Arabidopsis, nonstructural carbohydrates, such as sucrose, contribute at most 2% of the total osmotically active particles in the shoot in response to high salinity [76]. Those observations, together with our findings, suggest that any osmotic contribution from sucrose and hexoses as compatible osmolytes is limited.

### 3.2. Sugar Accumulation under High Salinity and Factors Acting in Their Storage in the Vacuole

Because sucrose and fructose increased in the stem of the salt-treated plants at 13 DABS, we measured the expression of genes controlling sugar partitioning (Figure 4). In salt-treated plants was observed an up-regulation of *TMT2*, coding for a tonoplastic transporter acting in the delivery of glucose, fructose and sucrose inside the vacuole [77,78]. By contrast, *SWEET2*, *SWEET16* and *SWEET17*, coding for facilitators that mediate glucose, fructose and/or sucrose transport across the tonoplast along the concentration gradient [79,80,81], were downregulated. These data support the hypothesis of a reduction of the cytosolic sugar towards storage in the vacuole (Figure 5). For *SWEET13* and *SWEET14*, encoding sugar and gibberellin facilitators in the plasma membrane of the vasculature [68,82], we observed contrasted responses to salt treatment, with *SWEET13* being downregulated and *SWEET14* upregulated. Interestingly, the expression of *SWEET14* was correlated to that of *P5CS1* (Supplementary Figure S2), a gene involved in the biosynthesis of proline and associated with the primary salt stress response [83], suggesting a common transcriptional regulatory pathway for these two genes.

Because fructokinases may also play an important role in the control of sugar homeostasis in sink tissues under changing conditions [84], we analyzed the expression of *FRK1*,*2*,*3*,*5*,*6* and *7* in response to salt treatment (Figure 5). *FRK1*, *2*, *3*, *5* and *7* were all downregulated (Figure 4). FRK proteins contribute to the homeostasis of cytosolic and plastidial fructose content by a conversion into Fructose 6P [85]. Overall, the transcriptional downregulation of *FRK1*,*2*,*3*,*5* and *7* and of *SWEET2*, *16* and *17* coding for tonoplastic sugar facilitators, the upregulation of *TM2*, coding for a tonoplastic sugar transporter and the high fructose content measured in the stem suggest a higher fructose storage in the vacuole under high salinity as well as sucrose. However, due to a poor understanding of the cellular expression patterns of these genes, except for *SWEET11*, *SWEET12*, *SWEET16* and SWEET17, for which a detailed picture of their expression pattern in the vascular parenchyma cells is available [86], it is not clear whether such changes in sugar partitioning occur in all cell types or are limited to cells in which Na^+^ ions are sequestered. In *Phaseolus vulgaris*, a natrophobic species, xylem parenchyma cells have been shown to accumulate the Na^+^ ions when plants were fed with sodium in the transpiration stream [87], but the cellular sites of sequestration of Na^+^ in the stem remain unknown in Arabidopsis. In addition, it is not clear whether these transcriptional responses are a result of direct regulation by Na^+^ accumulation or due to sensing of photoassimilates, since several of these genes have been described as regulated by sugars [53,88].

### 3.3. Modifications of Xylem Secondary Cell Wall Composition in the Stem

As well as affecting fructose levels, FRKs are also indirectly involved in the production of cell wall polysaccharides by feedback regulation of the SUSY activity and UDP-glucose production [89]. Under high salinity, we found for the stem that *SUSY3* expression was upregulated, while both *CwINV* and *CINV* were downregulated (Figure 4 and Figure 5). One could therefore expect changes in the cell wall composition as a consequence of the deregulation of *SUSY*, *CwINV*, *CINV* and *FRK* expression, as shown in other species [90,91,92]. Fourier-transform infrared spectroscopy showed that the xylem secondary cell wall of SStr plants exhibited relatively more cellulose and hemicelluloses (i.e., xylans) and less lignin (Figure 7).

A reduced thickening of interfascicular fibers, xylem vessels and fibers has been reported in the stem of Arabidopsis under salt stress [34]. Both that first report of the stem response [34] and our findings show that lignin deposition is not increased in the xylem of the inflorescence, unlike other abiotic stresses that trigger the reinforcement of the secondary cell wall with hemicellulose and lignin deposition [24,93]. As a result of lignin deficiency, xylem vessels are likely to be less able to withstand the higher xylem tensions caused by the raised soil osmotic pressure of a salt treatment [94,95]. This may explain the partial collapse of vessels that we observed, with irregular xylem cells. The modifications of cellulose and hemicelluloses content in the stems of salt treated plants may contribute to confer more elasticity to the cell wall to compensate to some extent for the lignin deficiency.

Interestingly, in acclimated PStr plants, lignin content was reduced, as observed in SStr plants, but different modifications in cellulose and hemicellulose contents were observed (Figure 7C), indicating a large plasticity in the composition of the secondary cell walls in polysaccharides under high salinity, that may be associated with the low number of irx cells observed in PStr plants compared to SStr ones. We recently showed that the irx phenotype observed in three Arabidopsis accessions is not associated with their tolerance to salt stress [35], indicating that changes in xylem secondary cell wall composition are likely not associated to an adaptive response to salt stress. Instead, they may reveal short-term changes in the prioritization of carbon pools in the stem to limit locally high consumption of carbon associated, for example, with lignification. Similarly, distinct effects were observed on the vascular area in SStr or Pstr plants, with a reduced xylem area per stem section and a thicker interfascicular zone in SStr plants, indicating that high salinity also impairs the development of the vascular tissues. It is not clear whether these anatomic modifications are a result of changes in sugar homeostasis or contribute to readjustment of sugar partitioning. What could be the long-term consequences of these adjustments in sugar homeostasis for this Arabidopsis sensitive accession remains to be explored. The latter might be an interesting issue to explore regarding consequences on later floral stem development, especially on silique formation and on seed yield, size and quality.

## 4. Materials and Methods

### 4.1. Plant Material and Growth Conditions

The Columbia accession of *Arabidopsis thaliana* (Col0) was used for the experiments. Col0 is considered as salt sensitive [96,97]. Seeds were surface sterilized then sown directly in soil. Plants were grown in a growth chamber with a long-day photoperiod (16 h light at 21 °C – 8 h dark at 17 °C) with an irradiance of 150 µmol·m^−2^·s^−1^ obtained with sodium lamps and 65% humidity. They were watered by immersion of the base of the pots in standard nutrient solution (10 mM Nitrate, 2.75 mM potassium, 0.5 mM calcium, 0.7 mM chloride, 0.25 mM phosphate) [98]. The floral bud emerged at 24 days after sowing, in comparison with 34 days for the plants grown by Sellami et al. [35] under different light intensity and spectrum.

### 4.2. Salt Treatment and Acclimation Experiments

For salt treatment (SStr), 150 mM of NaCl was added to standard nutrient solution at 23 days after sowing preceded or not by progressive pre-treatment (PStr) from 19 days after sowing, of 25 mM f of NaCl for two days, and 50 mM NaCl for two additional days, before the beginning of the 150 mM salt treatment. In these conditions, floral bud emergence occurred at 24 days after sowing, one day after the beginning of the salt treatment (DABS). Salt was applied by immersing pots in the 150 mM NaCl-nutrient solution for 6 h, three times per week for 2 weeks.

### 4.3. Growth Parameters

Projected rosette area (PRA) was measured from pictures taken at 4 and 11 DABS and using ImageJ Software (National Institute of Mental Health, Bethesda, MD, USA) [99]. The height of the stem was measured at 13 DABS.

### 4.4. Carbohydrate and Starch Content

For sugar and starch analysis, rosette leaves, including mature and young leaves were sampled at 4 DABS and 11 DABS and frozen in liquid nitrogen and stored at −80 °C until use. Mature leaves corresponded to the leaf numbers 7 to 12 that emerged before the beginning of salt treatment. Young leaves corresponded to leaf numbers 12 to 16 that emerged after beginning of salt stress. Main stems were sampled at 13 DABS and immediately frozen at −80 °C after removal of flowers and siliques and a section of the first basal cm that was used for the anatomical studies. Tissue was sampled six hours after the beginning of the light period. Soluble sugars were extracted from 50 mg of frozen powder by two successive additions of 80% ethanol kept for 2 h in ice. The supernatants were then separated from the residual solid material, evaporated with a speed-vac, and re-suspended in 50 µL of water. Sucrose and hexose content were determined from the ethanolic extract using an enzymatic sugar Kit (Sucrose/D-glucose/D-fructose kit, R-BioPharm, Germany). The pellet, kept for starch determination, was solubilized in water and incubated at 100 °C for six minutes. Starch content was determined using the sugar kit after release of glucose by incubation with α-amylase and γ-amylase (Boehringer Mannheim, Germany) at 50 °C for 3 h in 20 mM acetate buffer, pH 4.6 [98]. For each genotype, 4 biological replicates were analyzed per condition.

### 4.5. Proline Quantification

The free proline content was also measured on the extracts that were used for sugar quantification (see above) as described [100] with some modifications. Two hundred microliters of acid ninhydrin and 200 µL of glacial acetic acid were added to 20 µL of extract and 80 µL of sterile water and the reaction was started at 100 °C for 1 hour. The proline content of samples was calculated by comparison with a standard curve drawn from absorbance readings at 520 nm, using Spectro Star Nano ANAL Y0068955 (BMG Lab Tech, Champigny s/Marne, France), with a concentration range of proline in solution.

### 4.6. Anatomy of Stem Sections

The first cm of the basal part of the stem sampled at 13 DABS was embedded in 8% low-melting agarose. Transverse thin sections (50 µm thick) were cut on a Vibratome Zeiss MM France and stored in ethanol 70% at 4 °C. For imaging of the section, the sections were colored for 20 s with Safranin O-Alcyan Blue staining solution (1:1 *v*/*v*) diluted 1/10 with water [101] and observed by Zeiss Stereo Microscopy using color camera and transmitted light. The pictures were treated using ImageJ Software (bundled with 64-bit Java 1.8.0) by measuring the diameter of the transverse sections, the total area per cross section and the number of vascular bundles. Additionally, for each vascular bundle the total area of phloem and xylem were measured. The total area of xylem per cross section was calculated as the sum of individual xylem areas for each vascular bundle. The total lignified area per section corresponds to the interfascicular fibers plus xylem poles, visualized by lignin staining with safranin O. The proportion of lignified tissues was determined as the ratio of the total lignified area divided by the total area per cross section. For each xylem pole, the number of deformed xylem vessels was measured using the cells counter plugin of ImageJ (National Institute of Mental Health, Bethesda, MD, USA) [102]. Six to nine xylem poles were analyzed per section, with 5–6 biological replicates per condition.

### 4.7. Fourier-Transform Infrared Microspectroscopy

Half of the transverse cross-sections obtained as described above were used for FT-IR analysis. The samples were dried on BaF2 slides at 37 °C for 20 min. Spectra were collected on a Nicolet TM iN10 FT-IR microscope (Thermo Scientific, Courtaboeuf, France) from metaxylem as described [72]. The acquisition window of the microspectrophotometer was set up to 30 per 30 µm. Fifteen regions were analyzed per xylem pole. Between 90 and 248 spectra were acquired from two to four biological replicates per condition. All datasets were corrected for baseline and area-normalized using a homemade *R* script [103]. To test for the statistical significance of the differences, a modified Student *t*-test was performed using R software to do pairwise comparisons [103]. The *t*-values above +2 or below 2 correspond to significantly weaker and stronger absorbances.

### 4.8. RNA Extraction and qPCR Analysis

For RNA analysis, we used the mature rosette leaves sampled at 11 DABS and the main stem was sampled at 13 DABS. The samples were collected six hours after the beginning of the light period and immediately frozen in liquid nitrogen and stored at −80 °C until use. Total RNA was isolated from frozen tissue using TRIzol^®^ reagent (Invitrogen, Villebon-sur-Yvette, France). Reverse transcription was performed with 2 µg total RNA with the Superscript II enzyme (Invitrogen) after DNase treatment (Invitrogen). The primers used were either designed with the Primer3 software [104] or taken from literature (Supplementary Table S1 and S2). qPCR analysis was done on a Bio-Rad CFX96 Real-Time PCR machine and quantification of relative gene expression levels was done using the Bio-Rad CFX MANAGER 3.0 software [105]. Four reference genes were used, *APT1* (At1g27450), *TIP41* (At4g34270), *EF1α* (At5g60390) and *UBQ5* (At3g62250), with the most stable one *APT1* chosen using the geNorm algorithm [106] and selected for relative expressions. The relative expression level for each sample was calculated as described [105]. Average values were obtained from five to six biological replicates.

### 4.9. In Silico Analysis

Candidate genes were selected from the publicly available transcriptomic database ‘Bio-Analytic resource for Plant Biology’ (BAR) [107]. The expressions of candidate genes related to sugar transport and metabolism were extracted from the stress series datasets, for salt, drought, cold and heat stresses [47] applied on 16-day-old seedlings grown in liquid medium, in long day conditions [108]. In these experiments, a 150 mM NaCl salt treatment was applied on plants grown on liquid media. Data corresponding to the gene expression response after 24 h both in the roots and the shoots obtained after normalization by the control condition, were extracted from the database and then visualized on Genesis 1.8.1 software after log2 transformation [109]. Hierarchical clustering was done using the complete linkage option and the threshold for automatic gene cluster assignment was selected to obtain 12 clusters.

### 4.10. Statistical Analyses

Student’s *t* test was realized on Microsoft Excel software. One-way ANOVA combined with a Tukey’s comparison post-test was done using R software, version 3.3.2 [110]. A *p*_value_ <0.05 was considered as significant. Principal component analysis was performed using FactoMineR package of R [111]. Heat maps showing the response of each factor were constructed from the mean by hierarchical cluster analysis realized with Genesis 1.8.1 software after log2 transformation and normalization by the median [109]. Pearson correlations were realized using R with adjusted *p*-values calculated with the Holm’s method.

## Figures and Tables

**Table 1:** Comparison of sugar and starch contents in the rosette and leaves in response to salt stress.

Sample	Floral Stem			Mature Rosette Leaves			Young Rosette Leaves		
Treatment	Control	Salt	*t*-test	Control	Salt	*t*-test	Control	Salt	*t*-test
Soluble sugars nmol/mg FW	4.69 ± 0.57	8.66 ± 1.86	*	3.55 ± 0.31	3.99 ± 0.33	ns	4.87 ± 0.72	3.83 ± 0.34	ns
Hexoses/sucrose	2.05 ± 0.19	2.96 ± 0.46	ns	0.08 ± 0.01	0.01 ± 0.00	***	0.07 ± 0.00	0.02 ± 0.01	**
Sucrose/starch	4.53 ± 0.48	11.34 ± 1.31	**	1.08 ± 0.06	2.65 ± 0.29	*	0.94 ± 0.05	2.23 ± 0.40	**

ns: not significant. *t*-test: * *p* < 0.05; ** *p* < 0.01; *** *p* < 0.001.

## Supplementary Materials

Supplementary Materials can be found at https://www.mdpi.com/1422-0067/20/13/3167/s1.

## Abbreviations

DABS	Days after beginning of salt treatment
irx	irregular xylem phenotype
