# Supplementary material for: Salinity Effects on Sugar Homeostasis and Vascular Anatomy in the Stem of the Arabidopsis Thaliana Inflorescence
Source: Int J Mol Sci. 2019 Jun 28;20(13):3167. doi: 10.3390/ijms20133167 (PMC6651052; doi:10.3390/ijms20133167)
Supplement: Supplementary file 1 [file ijms-20-03167-s001.pdf]

International Journal of Molecular Sciences

MDPI

**Supplementary Table S1**. List of specific primers.

| Gene * | AGI | Forward primer | Reverse primer | Reference |
|---|---|---|---|---|
| **Stress markers** | | | | |
| *ERD6* | AT1G08930 | TGGCTGGACTGTGGAAGATT | CATCAACTGATTGGCGAACAC | [1] |
| *RD29A* | AT5G52310 | CTTGGCTCCACTGTTGTTCC | AATCCAAAGCCGAACAATTT | [2] |
| *P5CS1* | AT2G39800 | TTACCCCCAACAGTCTCTGG | GTTTTTGAATCCCGACCTGA | [3] |
| *P5CS2* | AT3G55610 | GCCACAAATGGAACTTGATGG | CAACCGTCACATCCAACTGGT | [3] |
| **Genes encoding sugar transporters** | | | | |
| *TMT2* | AT4G35300 | TGCTTCTCACCACGATACCA | AACCCATCACGAAGAAGCAG | This study |
| *SUC2* | AT1G22710 | TGCCTTTCACGATGACTGAG | TTCCTTGAAAGCTCCGAAGA | This study |
| *SUC4* | AT1G09960 | TCCCGTTGTATCTCCTCCTAGA | CAGCTCTTGAACGTAAGGTGTG | This study |
| *SWEET2* | AT3G14770 | AACAGAGAGTTTAAGACAGAGAGAAG | ATCCTCCTAAACGTTGGCATTGGT | [4] |
| *SWEET11* | AT3G48740 | TCCTTCTCCTAACAACTTATATACCATG | TCCTATAGAACGTTGGCACAGGA | [4] |
| *SWEET12* | AT5G23660 | AAAGCTGATATCTTTCTTACTACTTCG AA | CTTACAAATCCTATAGAACGTTGGCAC | [4] |
| *SWEET13* | AT5G50800 | CTTCTACGTTGCCCTTCCAAATG | CTTTGTTTCTGGACATCCTTGTTGA | [4] |
| *SWEET14* | AT4G25010 | ACTTCTACGTTGCGCTTCCAAATA | CAGTTCAACATTAAAGTCAATCACTAATTC | [4] |
| *SWEET15* | AT5G13170 | CAATGACATATGCATAGCGATTCCAA | GGACTCATCACGACAATACTCTTAAG | [4] |
| *SWEET16* | AT3G16690 | GAGATGCAAACTCGCGTTCTAGT | GCACACTTCTCGTCGTCACA | [4] |
| *SWEET17* | AT4G15920 | AGTGACAACAAAGAGCGTGAAATAC | ACTTAAACCGTTGCTTAAACCAACC | [4] |
| *G6PT/GPT2* | AT1G61800 | CGTAAGGCGGTCAATTCCTA | AACGTTAAGTGCCCACCAAG | This study |
| **Genes of the carbohydrate primary metabolism** | | | | |
| *CwINV 1* | AT3G13790 | CACATGTAAACACATTACATCTCCA | TTGGACAATTTTATTGACAACCA | This study |
| *CwINV 3* | AT1G55120 | TGTCTTCAACAAAGGCACTCA | CGTGACTCTTCACGCTCAAT | This study |
| *CINV1* | AT1G35580 | CACATGTAAACACATTACATCTCCA | TTGGACAATTTTATTGACAACCA | This study |
| *CINV2* | AT4G09510 | TGGTGTTCTTTCGTGGTCAA | TATCGGGCTCTCCATTCATC | This study |
| *SUSY1* | AT5G20830 | AGTGGTTCCGGTGTTTGAAG | TTCAAAGCAATGCCACAGAG | This study |
| *SUSY3* | AT4G02280 | GGTTCAGCGTTGGATGAAGT | CTCCATGGAAGATGGAACAAA | This study |
| *FRK1* | AT5G51830 | TCGCTCCTAAAATGCTTCAAA | CCGGGAGATCAACAACAAAC | This study |
| *FRK2* | AT2G31390 | CATTCCAGCTCTTCCCTCAG | CGATTCAACCATCCGAAAAC | This study |
| *FRK3* | AT1G66430 | CCTTGCTTCAGGACGAAGAG | CAGCTTCTTTGGTTGGAAGG | This study |
| *FRK5* | AT1G06020 | TTCGTTTGTTGGTGCACTTC | AGCTGGAATGGCTCCTTTTT | This study |
| *FRK6* | AT1G06030 | CTTTCCATGTTGACGCTGTG | CAAGCGTTTGCAAATCTCAG | This study |
| *FRK7* | AT3G59480 | TCAGAGCCTCCTGAAAGGAA | CCAAAAGCAGGGGAAAATAA | This study |
| **Reference genes** | | | | |
| *UBQ5* | AT3G62250 | CCAAGCCGAAGAAGATCAAG | ACTCCTTCCTCAAACGCTGA | [5] |
| *EF1α* | AT5G60390 | TGGTGACGCTGGTATGGTTA | TCCTTCTTGTCCACGCTCTT | [5] |
| *APT1* | AT1G27450 | GAGACATTTTGCGTGGGATT | CGGGGATTTTAAGTGGAACA | [5] |
| *TIP41* | AT4G34270 | GCTCATCGGTACGCTCTTTT | TCCATCAGTCAGAGGCTTCC | [5] |

* The function of these genes is described in Supplementary Table 2

## References

1. Wang, X.; Li, D.; Li, Q.; Ma, Y.; Yao, J.; Huang, X.; Xu, Z. Plant physiology and biochemistry metabolomic analysis reveals the relationship between AZI1 and sugar signaling in systemic acquired resistance of Arabidopsis. **2016**, *107*, 273–287.
2. Durand, M.; Mainson, D.; Porcheron, B.; Maurousset, L.; Lemoine, R.; Pourtau, N. Carbon source–sink relationship in Arabidopsis thaliana: the role of sucrose transporters. *Planta* **2018**, *247*, 587–611.

3. Alavilli, H.; Awasthi, J.P.; Rout, G.R.; Sahoo, L.; Lee, B.-H. Overexpression of a barley aquaporin gene, HvPIP2;5 confers salt and osmotic stress tolerance in yeast and plants. *Front. Plant Sci.* **2016**, *7*, 1–12.
4. Chen, L.-Q.; Hou, B.-H.; Lalonde, S.; Takanaga, H.; Hartung, M.L.; Qu, X.-Q.; Guo, W.-J.; Kim, J.-G.; Underwood, W.; Chaudhuri, B.; et al. Sugar transporters for intercellular exchange and nutrition of pathogens. *Nature* **2010**, *468*, 527–532.
5. Keech, O.; Pesquet, E.; Gutierrez, L.; Ahad, A.; Bellini, C.; Smith, S.M.; Per, G. Leaf senescence is accompanied by an early disruption of the microtubule network in Arabidopsis thaliana. *Plant Physiol.* **2010**, *154*, 1710–1720.
6. Toufighi, K.; Brady, S.M.; Austin, R.; Ly, E.; Provart, N.J. The Botany Array Resource: e-Northerns, Expression Angling, and Promoter analyses. *Plant J.* **2005**, *43*, 153–163.

**Supplementary Table S2.** List of candidate genes analyzed for *in silico* studies.

| Category | Name | AGI | Annotation |
|---|---|---|---|
| ***Stress Markers*** | | | |
| | *ERD6* | At1g08930 | ERD6_major facilitator superfamily protein |
| | *ESL1* | At1g08920 | ESL1_ERD (early response to dehydration) six-like 1 |
| | *P5CS1* | At2g39800 | P5CS1_delta1-pyrroline-5-carboxylate synthase 1 |
| | *P5CS2* | At3G55610 | P5CS2_delta1-pyrroline-5-carboxylate synthase 2 |
| | *RD29A* | At5g52310 | RD29A_ COR78_low-temperature-responsive protein 78 |
| | *RD29B* | At5g52300 | RD29B_ LTI65_CAP160 protein |
| ***Vascular Markers*** | | | |
| | *AHA3* | At5g57350 | AHA3_H(+)-ATPase 3 |
| | *CCH* | At3g56240 | CCH_copper chaperone |
| | *CCR2* | At1g80820 | CCR2_cinnamoyl CoA reductase |
| | *MT3* | At3g15353 | MT3_metallothionein 3 |
| | *PP2A1* | At4g19840 | PP2-A1_phloem protein 2-A1 |
| **Metabolism** | | | |
| | *AMY1* | At4g25000 | AMY1_alpha-amylase-like |
| | *AMY2* | At1g76130 | AMY2_alpha-amylase-like 2 |
| | *BAM1* | At3g23920 | BAM1_ BMY7_TR-BAMY_beta-amylase 1 |
| | *BAM5* | At4g15210 | BETA-AMY_BAM5_BMY1_RAM1_beta-amylase 5 |
| | *BAM9* | At5g18670 | BAM9_BMY3_beta-amylase 3 |
| | *ADG2* | At5g19220 | ADG2_ADP glucose pyrophosphorylase large subunit 1 |
| | *A/N-InvB* | At4g34860 | A/N-InvB_plant alkaline/neutral invertase (predicted cytosolic) |
| | *A/N-InvC* | At3g06500 | A/N-InvC_ plant alkaline/neutral invertase (mitochondrial) |
| | *A/N-InvH* | At3g05820 | A/N-InvH, INVH alkaline/neutral invertase H (mitochondrial) |
| | *CINV1* | At1g35580 | A/N-InvG_CINV1 cytosolic invertase 1 |
| | *CINV2* | At4g09510 | A/N-InvI_CINV2_cytosolic invertase 2 |
| | *CwINV2* | At3g52600 | CWINV2_cell wall invertase 2 |
| | *CwINV4* | At2g36190 | CWINV4_cell wall invertase 4 |
| | *CwINV5* | At3g13784 | CWINV5_cell wall invertase 5 |
| | *FRK1* | At5g51830 | pfkB-like carbohydrate kinase family protein FRK1 |
| | *FRK2* | At2g31390 | pfkB-like carbohydrate kinase family protein FRK2 |
| | *FRK3* | At1g66430 | pfkB-like carbohydrate kinase family protein FRK3 |
| | *FRK4* | At4g10260 | pfkB-like carbohydrate kinase family protein FRK4 |
| | *FRK5* | At1g06020 | pfkB-like carbohydrate kinase family protein FRK5 |
| | *FRK6* | At1g06030 | pfkB-like carbohydrate kinase family protein FRK6 |
| | *FRK7* | At3g59480 | pfkB-like carbohydrate kinase family protein FRK5 |
| | *GolS6* | At4g26250 | GolS6_galactinol synthase 6 |
| | *SPS1F* | At5g20280 | SPS1F_SPSA1_sucrose phosphate synthase 1F |
| | *SUSY1* | At5g20830 | SUS1_sucrose synthase 1 |
| | *SUSY2* | At5g49190 | SUS2_sucrose synthase 2 |
| | *SUSY3* | At4g02280 | SUS3_sucrose synthase 3 |
| | *SUSY4* | At3g43190 | SUS4_sucrose synthase 4 |
| | *SUSY5* | At5g37180 | SUS5_sucrose synthase 5 |

| | | | |
|---|---|---|---|
| | *SUSY6* | At1g73370 | SUS6_sucrose synthase 6 |
| | *UGD1* | At1g26570 | UGD1_UDP-glucose dehydrogenase 1 |
| | *UGD2* | At3g29360 | UGD2_UDP-glucose 6-dehydrogenase family protein |
| | *UGD3* | At5g15490 | UGD3_UDP-glucose 6-dehydrogenase family protein |
| | *UGT1* | At1g05560 | UGT1_UGT75B1_UDP-glucosyltransferase 75B1 |
| | *UGT72E1* | At3g50740 | UGT72E1_UDP-glucosyl transferase 72E1 |
| | *UGT72E2* | At5g66690 | UGT72E2_UDP-glycosyltransferase superfamily protein |
| | *UGT72E3* | At5g26310 | UGT72E3_UDP-glycosyltransferase superfamily protein |
| | *CalS1* | At1g05570 | GSL06_ CALS1_callose synthase 1 |
| | *CalS3* | At5g13000 | GSL12_ CALS3_glucan synthase-like 12 |
| | *CalS5* | At2g13680 | GLS02_ CALS5_callose synthase 5 |
| | *CalS7* | At1g06490 | GSL07_ CALS7_glucan synthase-like 7 |
| | *GSL1* | At4g04970 | GSL01_ glucan synthase-like 1 |
| | *GSL3* | At2g31960 | GSL03_glucan synthase-like 3 |
| | *GSL4* | At3g14570 | GSL04_glucan synthase-like 4 |
| | *GSL5* | At4g03550 | GSL05_ EED3_PMR4_glucan synthase-like 5 |
| | *GSL9* | At5g36870 | GSL09_ glucan synthase-like 9 |
| | *GSL11* | At3g59100 | GSL11_glucan synthase-like 11 |
| **Signaling** | | | |
| | *HXK3* | At1g47840 | HXK3_hexokinase 3 |
| | *SNRK2.2* | At3g50500 | SNRK2.2_SRK2D_SNF1-related protein kinase 2.2 |
| | *SNRK2.3* | At5g66880 | SNRK2.3_SRK2I_SNF1-related protein kinase 2.3 |
| **Transport** | | | |
| | *INT2* | At1g30220 | INT2_inositol transporter 2 |
| | *INT3* | At2g35740 | INT3_inositol transporter 3 |
| | *G6PT* | At1g61800 | *G6PT_GPT2_* glucose-6-phosphate/phosphate translocator 2 |
| | *PMT5* | At3g18830 | PLT5_PMT5_polyol/monosaccharide transporter 5 |
| | *STP4* | At3g19930 | STP4_sugar transporter 4 |
| | *STP7* | At4g02050 | STP7_sugar transporter protein 7 |
| | *STP11* | At5g23270 | STP11_sugar transporter 11 |
| | *STP12* | At4g21480 | STP12_sugar transporter protein 12 |
| | *STP13* | At5g26340 | STP13_MSS1_ major facilitator superfamily protein |
| | *STP14* | At1g77210 | STP14_sugar transporter 14 |
| | *SUC2* | At1g22710 | SUC2_SUT1_sucrose-proton symporter 2 |
| | *SUC3* | At2g02860 | SUC3_SUT2_sucrose transporter 2 |
| | *SUC4* | At1g09960 | SUC4_SUT4_sucrose transporter 4 |
| | *SUC5* | At1g71890 | SUC5_major facilitator superfamily protein |
| | *SUC6* | At5g43610 | SUC6_sucrose-proton symporter 6 |
| | *SUC7* | At1g66570 | SUC7_sucrose-proton symporter 7 |
| | *SUC8* | At2g14670 | SUC8_sucrose-proton symporter 8 |
| | *SUC9* | At5g06170 | SUC9_sucrose-proton symporter 9 |
| | *SWEET1* | At1g21460 | SWEET1_nodulin MtN3 family protein |
| | *SWEET2* | At3g14770 | SWEET2_ nodulin MtN3 family protein |
| | *SWEET3* | At5g53190 | SWEET3_ nodulin MtN3 family protein |

| | | |
|---|---|---|
| *SWEET4* | At3g28007 | SWEET4_ nodulin MtN3 family protein |
| *SWEET8* | At5g40260 | SWEET8_RPG1_ nodulin MtN3 family protein |
| *SWEET11* | At3g48740 | SWEET11_ nodulin MtN3 family protein |
| *SWEET12* | At5g23660 | SWEET12_ nodulin NtN3 family protein |
| *SWEET13* | At5g50800 | SWEET13_ nodulin MtN3 family protein |
| *SWEET14* | At4g25010 | SWEET14_ nodulin MtN3 family protein |
| *SWEET15* | At5g13170 | SWEET15_ SAG29_senescence-associated gene 29 |
| *SWEET16* | At3g16690 | SWEET16_ nodulin MtN3 family protein |
| *SWEET17* | At4g15920 | SWEET17_ nodulin MtN3 family protein |
| *TMT1* | At1g20840 | TMT1_tonoplast monosaccharide transporter1 |
| *TMT2* | At4g35300 | TMT2_tonoplast monosaccharide transporter2 |

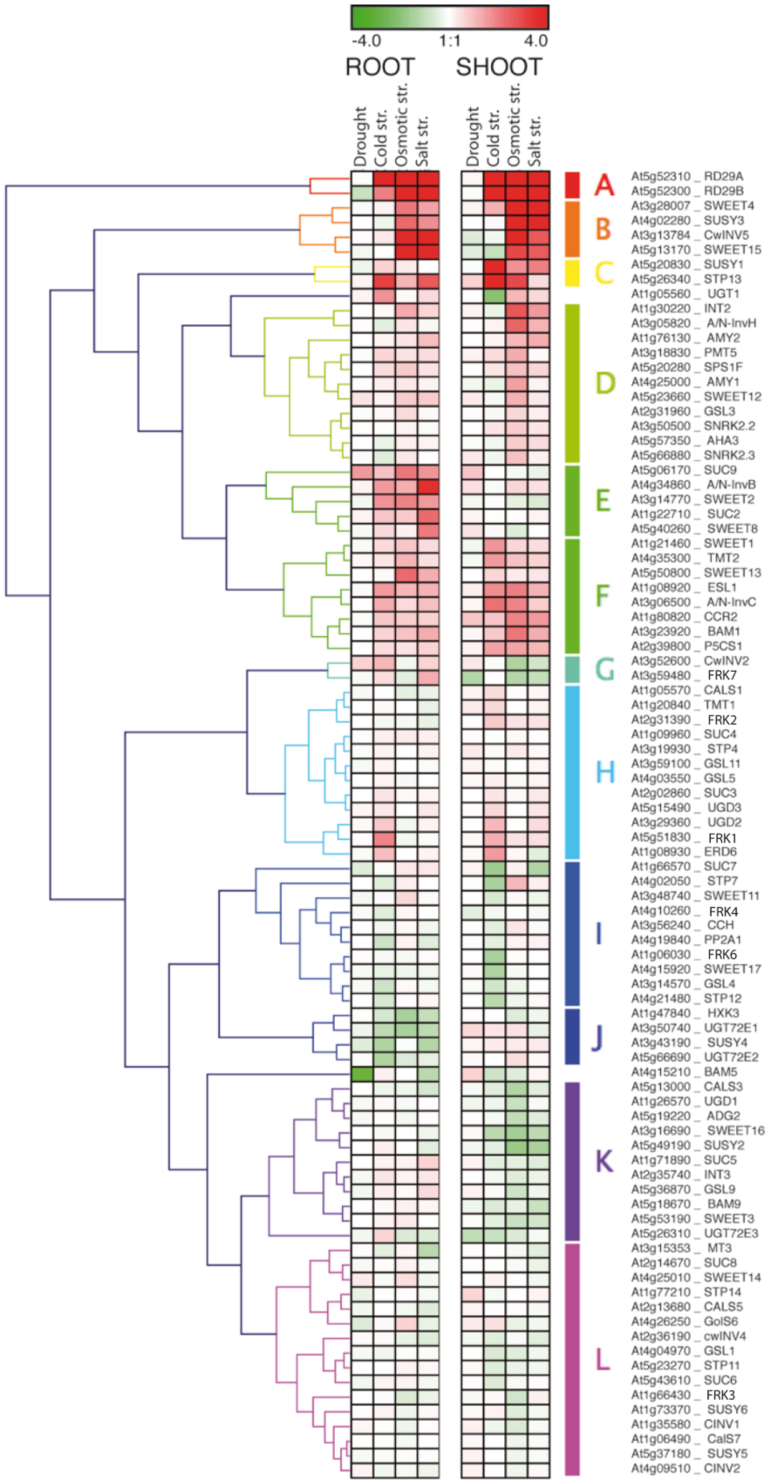


**Supplementary Figure S1**. *In silico* analysis of transcript profiling of candidate genes in the roots and shoots of Col0 plants. The list of genes and their function is described in the Supplementary Table 2. The relative accumulation of transcripts for each candidate gene was determined *in silico* on Bio-Analytic resource – Expression Browser using the stress series database [6]. The data correspond to the response of 18-day-old plants grown *in vitro* and submitted for 24 hr to drought (first column), cold stress (second column), osmotic stress (third column) or salt stress (last column). Str.: stress. On the left side are reported the responses observed in the roots and on the right side the responses observed in the shoot. The values correspond to log2-transformed ratios of stress plants versus control plants, with green color scale showing lower accumulation and red color scale showing higher accumulation than controls. Several subgroups obtained by automatic clustering are shown on the right side of the figure.

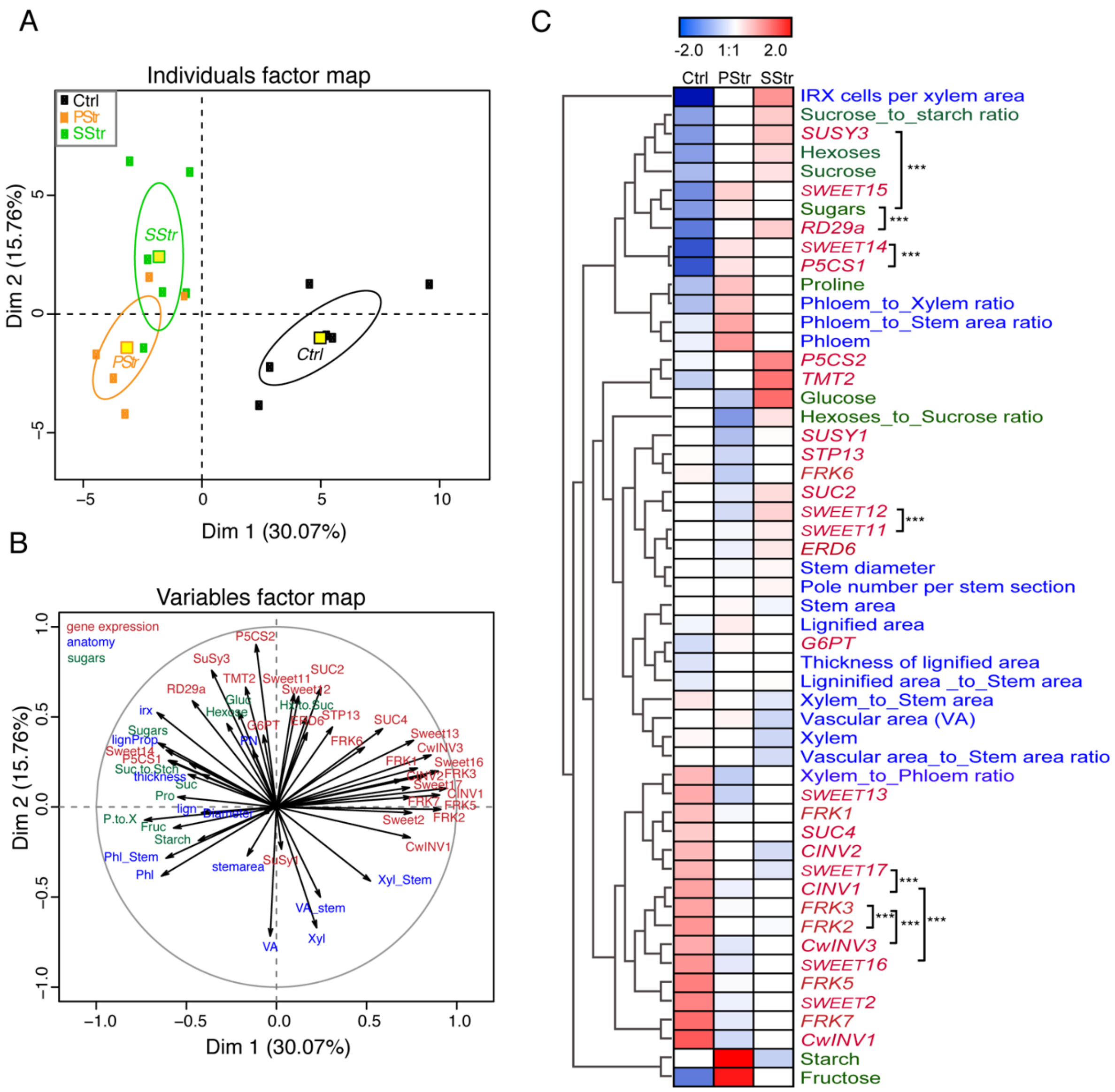


**Supplementary Figure S2.** Principal component analysis and hierarchical cluster analysis of anatomical, physiological and transcriptomic factors measured in the stem in Ctrl, SStr and PStr plants. The factors are: 1) Transcript profiles relative to stress markers, sugar transporters and sugar metabolism, 2) Sugars, starch and proline contents (in nmole per mg of fresh weight): Fruc, Gluc, Suc, total soluble sugars (Sugars), starch, hexoses, hexoses-to-sucrose ratio (Hx.to.Suc), sucrose-to-starch ratio (Suc.to.Stch) and proline (Pro). 3) Anatomical factors: stem section area (stem area, in mm$^2$), lignified area in the stem section (lign, in mm2); thickness of the lignified area (thickness), proportion of the lignified area in the main stem (lignProp); diameter of the main stem (diameter, in mm), pole number (PN); total xylem area in the stem (Xyl, in in mm$^2$), total phloem area in the stem (Phl, in mm$^2$), xylem area-to-phloem area ratio (X.to.P), vascular area (VA, in mm$^2$), xylem area-to-stem area ratio (Xyl_Stem), Phloem area -to-Stem area ratio (Phl_Stem), vascular area-to-Stem area ratio (VA_stem), number of irregular xylem cells per xylem area (irx, number per µm$^2$), phloem area-to-xylem area ratio (P.to.X). In (A) and (B) PCA showing the projection on the 2 first components of the samples (A) and factors (B). In (A) control plants are shown in black, PStr plants in red and SStr plants in green. In (B) Transcritomic factors are drawn in red, anatomical factors in blue and sugars and proline contents in green. (C) Heat map obtained after hierarchical cluster analysis visualizing the mean values for each factor drawn after log2 transformation and normalization by the median ($n$=4-6). We used the same color code as in (B). Asterisks indicate significant Pearson correlations between factors (*: $p < 0.05$; **: $p < 0.01$; ***:$p < 0.001$).
